# Cecropin A Improves the Antibacterial Activity of Hen Egg White Lysozyme against Challenging *Salmonella enterica* Serovars

**DOI:** 10.3390/pharmaceutics14102201

**Published:** 2022-10-16

**Authors:** Hani A. Alhadrami, Ahmed M. Sayed, Hossam M. Hassan, Mostafa E. Rateb, Karim Abdelkader

**Affiliations:** 1 Department of Medical Laboratory Technology, Faculty of Applied Medical Sciences, King Abdulaziz University, P.O. Box 80402, Jeddah 21589, Saudi Arabia; hanialhadrami@kau.edu.sa; 2 Molecular Diagnostic Laboratory, King Abdulaziz University Hospital, King Abdulaziz University, P.O. Box 80402, Jeddah 21589, Saudi Arabia; 3 King Fahd Medical Research Center, King Abdulaziz University Hospital, King Abdulaziz University, P.O. Box 80402, Jeddah 21589, Saudi Arabia; 4 Department of Pharmacognosy, Faculty of Pharmacy, Nahda University, Beni-Suef 62513, Egypt; ahmed.mohamed.sayed@nub.edu.eg (A.M.S.); hossam.mokhtar@nub.edu.eg (H.M.H.); 5 Department of Pharmacognosy, Faculty of Pharmacy, Beni-Suef University, Beni-Suef 62513, Egypt; 6 School of Computing, Engineering & Physical Sciences, University of the West of Scotland, Paisley PA1 2BE, UK; 7 Department of Microbiology and Immunology, Faculty of Pharmacy, Beni-Suef University, Beni-Suef 62511, Egypt

**Keywords:** *Salmonella enterica*, hen egg white lysozyme, cecropin A, amphipathic helix, food preservative

## Abstract

The prevalence of multidrug-resistant *Salmonella enterica* among animal- and plant-derived food products threatens global healthcare and economic sectors. Hen egg white lysozyme is widely exploited as a food preservative against Gram-positive pathogens. Nevertheless, its limited penetration of the outer membrane renders it ineffective against Gram-negative bacteria. Herein, we present a safe and effective approach to facilitate HEWL access to peptidoglycan layers using cecropin A. In silico analysis of cecropin A peptide revealed an amphipathic α-helical peptide with potential outer membrane permeabilizing activity through its interaction with both hydrophobic and ionic stabilizing forces. Evaluation of HEWL/cecropin A combination showed a cecropin A dose-dependent bacterial count reduction up to 4.16 and 3.18 ± 0.26 log units against *Salmonella enterica* ATCC 35664 at the logarithmic and stationary growth phases, respectively. Moreover, the combination displayed antibacterial activity of 2.1 ± 0.31 and ~1 log-unit reductions against *Salmonella enterica* serovars Kentucky, Typhimurium, and Enteritidis, respectively, whereas Hato and Shangani were found irresponsive. The cytotoxicity assay revealed compatibility of cecropin A with oral epithelial cells. These observations suggest HEWL/cecropin A combination as an effective and safe alternative to lysozyme against *Salmonella enterica*.

## 1. Introduction

The unprecedented spread of multidrug-resistant bacteria poses a real burden on both the healthcare and economic sectors. Gram-negative bacteria are particularly challenging due to their reported natural and acquired resistance to different classes of traditional antibiotics [1]. The limited permeability of the Gram-negative bacterial outer membrane is the major contributor to their natural resistance to a multitude of antibacterial agents (penicillin, cephalosporins, lysozymes, and phage lysins). *Salmonella* spp. is a leading Gram-negative food pathogen threatening the food industry, animal production, and human health [2]. Globally, approximately 94 million foodborne illnesses and over 150,000 deaths have been reported due to *Salmonella* spp. infection [3]. In the USA, an economic loss of USD 400 million has been estimated as consequence of its infection [4]. In particular, *Salmonella enterica* is the most problematic species with more than 2600 serovars [5]. Most of these serovars can infect and reside within different animal hosts and humans, making their dissemination easier upon contact [6]. The treatment of salmonella infections is challenging due to the emergence of multidrug-resistant strains [7,8]. Therefore, finding alternative approaches is imperative to control this growing problem.

Peptidoglycan hydrolases (PGHs) are diverse enzymes working on the peptidoglycan component of the bacterial cell wall. Upon their exogenous application, specific chemical bonds in the peptidoglycan meshwork are degraded, making bacteria more prone to osmotic lysis [9]. Unlike traditional antibiotics, PGHs do not target specific metabolic pathways to inhibit bacterial growth; alternatively, they work on the conserved peptidoglycan [10]. Therefore, they have a relatively faster action, less dependence on the bacterial growth phase, and a low probability of being resisted [10]. Lysozymes are a natural, abundant, and ubiquitous class of PGHs that specifically cut the 1–4 β-glycosidic linkage between N-acetylmuramic acid and N-acetyl-β-glucosamine residues in the peptidoglycan meshwork. Lysozymes are present as a major component of animal products (egg white, hen egg white lysozyme) and are also found in body fluids (tears, saliva, and respiratory secretions) as an important contributor to innate immunity [11]. They are widely used as food preservatives to inhibit bacterial growth in food products leading to a longer shelf life. The antibacterial activity of lysozymes was reported in both pharmaceutical and food fields against Gram-positive bacteria [12]. Nonetheless, their activity is largely diminished in the case of Gram-negative bacteria due to the protective outer membrane barrier [13]. Many efforts have been employed to expand the antibacterial activity of the lysozyme to cover Gram-negative bacteria, including the addition of outer membrane permeabilizers [14] (e.g., EDTA), application of high hydrostatic pressure [15], enzyme denaturation [16], and dextran conjugation [17]. However, most of these approaches may limit lysozyme application due to systemic toxicity (e.g., EDTA) or higher costs (high hydrostatic pressure and dextran conjugation). Moreover, Salmonella species have been shown to be less sensitive to the outer membrane permeabilizing activity of EDTA due to the predominance of hydrophobic stabilizing forces between lipid A components of their outer membranes [18,19].

Antimicrobial peptides (AMPs) are a class of short peptides possessing outer membrane permeabilizing properties through interference with stabilizing ionic forces (established via the interaction of divalent cations with phosphate residues), hydrophobic forces (due to stacking of lipid A components), or both [20]. Cecropins are natural antimicrobial peptides derived mainly from insects with reported outer membrane permeabilizing activity. Structurally, cecropins (cecropins A, B, and D) are characterized by their positively charged N- terminal and neutral C-terminal that are linked together via a flexible glycine–proline link. This distribution of amino acids implies an amphipathic nature to cecropins, facilitating their interaction with both hydrophobic and ionic forces stabilizing the Gram-negative outer membrane [21,22]. Different studies have exploited the outer membrane permeabilizing activity of cecropins to facilitate the access of exogenously applied PGHs to the peptidoglycan layer [23,24] making the application of this class of enzyme-based antibiotics possible against Gram-negative bacteria. However, studying the combinatorial effect of AMPs with natural lysozymes for combating problematic food pathogens is still under-explored and thus merits further investigation.

In the current study, we have presented a new approach for extending the antibacterial activity of hen egg white lysozyme to cover the Gram-negative bacteria, *Salmonella enterica.* For this, we performed an in-depth in silico analysis to predict the full 3D structure of cecropin A and anticipate its possible interference with the outer membrane stabilizing forces to perform the reported outer membrane stabilizing activity [25]. Then, we investigated whether this activity could re-sensitize the different naturally lysozyme-resistant MDR *Salmonella enterica* serovars to the natural, abundant, and cheap hen egg white lysozyme. Moreover, the possible cytotoxicity of cecropin A was also investigated using the oral epithelial normal cell line.

## 2. Materials and Methods

### 2.1. Bacterial Strains and Culture Conditions

*Salmonella enterica* ATCC 35664 in addition to five *Salmonella enterica* serovars isolated from commercial poultry products [8] were included in the current study (Table 1). Lyophilized *Micrococcus lysodeikticus* ATCC 4689 (Sigma-Aldrich, St. Louis, MO, USA) was used as a positive control to optimize lysozyme concentration. Fresh bacterial cultures were prepared by transferring 100 µL of the strain glycerol stock (or one loop of lyophilized of M. lysodeikticus) into 5 mL LB (lysogeny broth; Himedia, India) and incubated at 37 °C for 24 h while shaking (200 rpm). The prepared cultures were used to streak LB agar (Himedia, India) plates (supplemented with 1.5% agar) that were incubated at 37 °C for 18–20 h. Then, the plates were stored in the fridge (up to 10 days) till they were used.

### 2.2. Cecropin A Preparation

Cecropin A (accession number AF117886.1) is a 36-amino acid peptide with a sequence of GGLKKLGKKLEGAGKRVFNAAEKALPVVAGAKALRK, here designed as CecA. The peptide was synthesized by Genescript (Leiden, Netherlands) and provided as lyophilized powder with a final purity of (≥95%). CecA stock solution (final concentration of 400 µg/mL) was prepared by adding 10 mM potassium phosphate buffer (pH 7.4), then aliquoted, and stored in −20 °C till use. For assays, the aliquot was thawed and diluted using 10 mM potassium phosphate buffer (pH 7.4). HEWL (Thermo Fisher Scientific, Waltham, MA, USA) was prepared as a stock of 10 mg/mL by mixing 10 mg HEWL in 1 mL 10 mM potassium phosphate buffer pH 7, then mixing gently till complete dissolution. The stock was kept in the fridge till use (up to 7 days).

### 2.3. In Silico Analysis and Modeling

#### 2.3.1. Cecropin A Model Preparation

We used the Alphafold-2 deep-learning-based algorithm to generate inter-residue distance predictions and to construct preliminary models [26,27,28,29,30]. We constructed 10 models for each protein, and the structure with the lowest energy was chosen for further refinement. Subsequently, the selected model was refined by running a set of molecular dynamics simulations [27]. In general, we followed the iterative protocol we had previously published [28], but without iterations. At 310 K, we conducted 5 trajectories and 50 ns-long simulations. We used RWplus [28] during the scoring phase.

#### 2.3.2. CecA-Membrane Model Preparation and Molecular Dynamics Simulation

The CecA-membrane system was constructed using the membrane builder in Charmm-GUI (https://www.charmm-gui.org/?doc=input/membrane.bilayer; accessed on 13 August 2022); [31]. The membrane mimics the outer membrane of *E. coli* and contains Ra LPS in the outer leaflet and 18:1:1 ratio of POPE, POPG, and CDL2 in the inner leaflet [29,30]. The orientation of the modeled cecropin within the constructed bilayer was predicted by the online platform (https://proteinformatics.uni-leipzig.de/rhythm/; accessed on 13 August 2022) [32]. Finally, the modeled system was loaded in NAMD 2.14 using the VMD graphical user interface and then simulated for 50 ns after the addition of modeled water molecules and 0.15 M of modeled Na^+^ and Cl^-^ ions. The generated output files were used to extract the RMSDs.

### 2.4. Minimum Inhibitory Concentration and Synergy Testing

Minimum inhibitory concentrations of CecA and Hen egg white lysozyme (HEWL, Thermo Fisher Scientific, Waltham, MA, USA; prepared as 10 mg/mL in 10 mM potassium phosphate buffer pH 7) were screened using a conventional broth microdilution assay [33]. CecA and HEWL stock solutions were diluted in Meuller Hinton broth (Thermo Fisher Scientific, Waltham, MA, USA) to obtain final concentration ranges of 2–20 µg/mL and 2–1000 µg/mL, respectively, then dispensed in the wells of a 96-well microtiter plate. Afterward, the exponentially grown (O.D600 of 0.6) test strains (*Salmonella enterica* ATCC 35664) in MH were diluted to 10^6^ CFU/mL and added to CecA and HEWL. The plates were then incubated without shaking at 37 °C for 18 h. A separate MIC experiment was conducted by challenging exponentially grown *M. lysodeikticus* ATCC 4689 with the HEWL concentration range of 2–1000 µg/mL as a positive control. Synergy testing between CecA and HEWL was performed using a checkerboard assay as described previously [34,35,36].

### 2.5. In Vitro Antibacterial Activity Assay of the Lysozyme and Cecropin A against Different Growth Phase Bacteria

Antibacterial activity of HEWL alone and in combination with CecA was conducted against exponentially and stationary grown *Salmonella enterica* ATCC 35664 as previously described [23]. Overnight culture of *Salmonella enterica* ATCC 35664 was used as bacteria in the stationary growth phase, whereas the logarithmic growth phase culture was prepared by 100-fold dilution of the overnight culture in fresh LB media followed by incubation at 37 °C till final OD of 0.6. For the antibacterial assay, the prepared cultures (both in exponential and stationary phases) were pelleted (12,000× *g* for 5 min), washed twice using 10 mM potassium phosphate buffer (pH 7.4) buffer, and diluted to a final inoculum density of ~10^6^ CFU/mL. Afterwards, the prepared cells were mixed with HEWL (final concentrations of 100–1000 µg/mL) and HEWL/CecA combination (final concentrations of 500 µg/mL HEWL with 0.5, 2, 4 µg/mL cecropin A). Parallel controls were conducted using CecA (using final concentrations of 0.5, 2, 4 µg/mL). The mixture was then incubated for 1 h at 25 °C without shaking. Fifty microliters of the mixture were serially diluted in a 10 mM potassium phosphate buffer (pH 7.4), plated on LB agar plates, and incubated for 18 h at 37 °C. Control was conducted by incubating the prepared bacterial cells with 10 mM potassium phosphate buffer (pH 7.4). Each experiment was performed as three independent replicates. A similar assay was conducted against other *Salmonella enterica* serovars (Table 1) that were grown to the exponential growth phase.

To investigate whether the CecA pretreatment could sensitize the exponentially grown *Salmonella enterica* ATCC 35664 to HEWL, the bacterial cells were exposed to sequential treatments of CecA and HEWL. Briefly, *Salmonella enterica* ATCC 35664 at the exponential growth phase (O.D of 0.6) was pelted (12,000× *g* for 5 min at 4 °C), washed twice using 10 mM potassium phosphate buffer, then 100-fold diluted in the same buffer. The prepared cells were then incubated with Cec A (final concentrations of 0.5 and 1 µg/mL) for 10 min, washed twice using 10 mM potassium phosphate buffer, and subsequently treated with 500 µg/mL HEWL for 1 h. Bacterial counts of the different treatment were measured as mentioned before. Controls were conducted by treating cells with 10 mM potassium phosphate buffer in place of CecA.

### 2.6. Cytotoxicity Assay of Cecropin A against Oral Epithelial Cells

Oral epithelial cells (OEC) were obtained from Nawah Scientific Inc., (Mokatam, Cairo, Egypt). Cells were maintained in Dulbecco’s Modified Eagle Medium (DMEM; Thermo Fisher Scientific, Waltham, MA, USA) supplemented with 100 mg/mL of streptomycin (Thermo Fisher Scientific, Waltham, MA, USA), 100 units/mL of penicillin and 10% of heat-inactivated fetal bovine serum. The cells were then incubated in a humidified incubator with 5% (*v*/*v*) CO_2_ atmosphere at 37 °C.

Cell viability was assessed by Sulforhodamine B (SRB) assay as described previously [37,38]. Aliquots of 100 μL cell suspension (5 × 10^3^ cells) were dispended in 96-well plates and incubated for 24 h. Cells were treated with another aliquot of 100 μL media containing CecA with final concentration range of 40–2.5 µg/mL and incubated for 72 h. Subsequently, the cells were fixed by replacing media with 150 μL of 10% trichloroacetic acid (TCA; Thermo Fisher Scientific, Waltham, MA, USA) and incubated at 4 °C for 1 h. The TCA solution was removed, and the cells were washed 5 times with distilled water. Aliquots of 70 μL SRB solution (0.4% *w*/*v*) were added and incubated in a dark place at room temperature for 10 min. Then the plates were washed 3 times with 1% acetic acid and allowed to air-dry overnight. Afterwards, 150 μL of TRIS (10 mM) was added to dissolve protein-bound SRB stain; the absorbance was measured at 540 nm using a BMGLABTECH^®^-FLUOstar Omega microplate reader (Ortenberg, Germany).

### 2.7. Statistical Analysis

A Student’s *t* test was conducted to differentiate between treated and untreated bacterial cells. Statistically significant difference was considered when *p* < 0.05. Statistical analysis was performed using SPSS Statistics for Windows v. 22.0 (IBM Corp., Armonk, NY, USA).

## 3. Results

### 3.1. In Silico Analysis of Cecropin A

**Figure 1:**
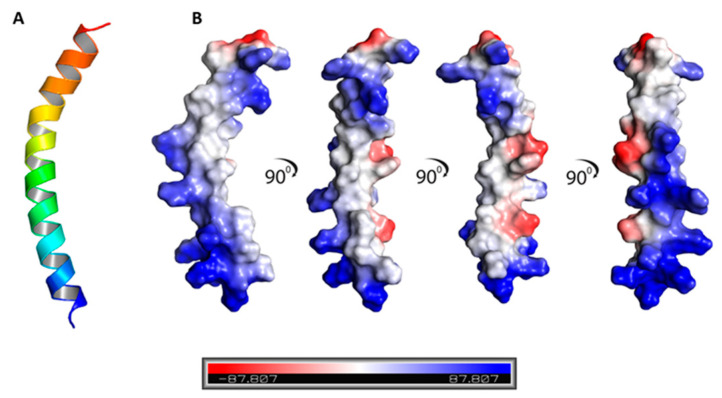
(**A**) Predicted 3D structure of the cecropin A peptide using AlphaFold-2. The predicted structure adopted an α-helix configuration. (**B**) Surface analysis of the predicted structure revealed alternate distribution of the charged amino acids (positively charged amino acids, blue, and negatively charged amino acids, red) and hydrophobic amino acids creating an amphipathic feature.

Based on previous studies underscoring the potential interaction of CecA with the outer membrane component of Gram-negative bacteria [23,24,39], we performed an in-depth in silico analysis for the CecA structure to investigate this hypothesis. The CecA primary amino acid sequence revealed a 36-amino acid peptide with a theoretical mass of 3675 Da and a predictive isoelectric point of 10.8. The included amino acids displayed different physiochemical properties with eight positively (Lysine and Arginine) and two negatively (Glutamate) charged amino acids, whereas the majority of the present amino acids were hydrophobic/non-polar (Glycine, Alanine, Phenylalanine, Valine; 15 amino acid). Protein structural analysis revealed an α-helical peptide of acceptable quality (Figure S1) displaying a net positively charged surface (Figure 1). Interestingly, the charged and non-charged amino acids were distributed alternatively on both sides of CecA peptide, creating an amphipathic nature (Figure 1 and Figure S2).

**Figure 2:**
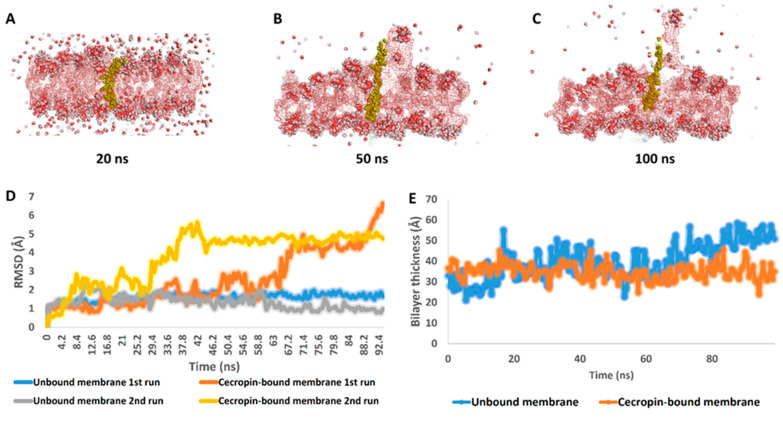
Snapshots extracted from the 100 ns-long simulation at 20 ns, 50 ns, and 100 ns (**A**–**C**). (**D**) Root Mean Square Deviations (RMSDs) of the bilayer system (CecA-bound and unbound) over two runs of 100 ns-long molecular dynamics simulations. (**E**) The average of the bilayer thickness changed (CecA-bound and unbound) over two runs of 100 ns-long MDS runs. The supplementary video can be found here: https://zenodo.org/record/7058606#.YzNGQHbP2M8, accessed on 28 August 2022.

The predicted amphipathic nature of CecA suggests its interaction with negatively charged phosphate residues and lipid A hydrophobic moieties of Gram-negative bacteria. These putative interactions were visualized by running a protein–membrane system simulation, which was subjected to a 50 ns-long MDS run. We found that the cecropin-bound membrane system was significantly distorted at the end of the simulation (RMSD ~ 14 Å) (Figure 2C,D) in comparison with the unbounded one (RMSD = 1.84 Å) (Figure 2D). This observation indicated that the amphipathic feature of a single CecA α-helix negatively affected the integrity of bacterial membranes. As previously discussed, this effect might arise from the amphipathic character of CecA. This, in turn, would help HEWL to penetrate the disrupted membrane and exert its antibacterial effect.

### 3.2. Cecropin A Expands the Antibacterial Spectrum of Lysozyme

**Figure 3:**
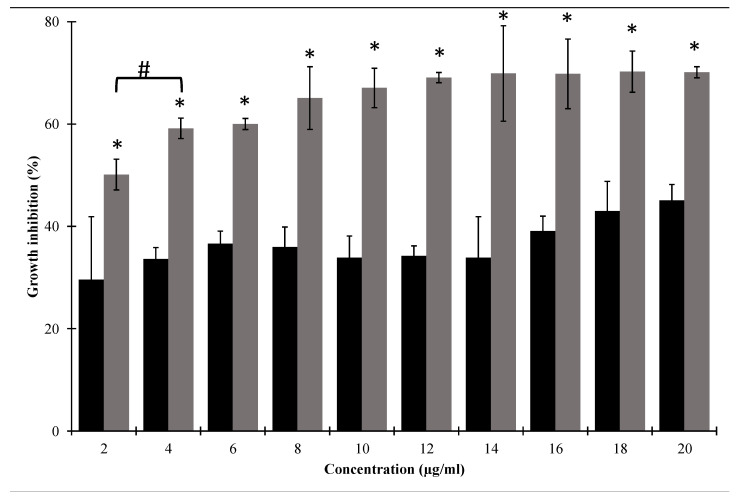
Growth inhibition analysis of combining HEWL with cecropin A (grey bars). Controls were conducted using cecropin A alone (black bars). The bars represent the growth inhibition activity of 500 µg/mL of HEWL combined with cecropin A concentration range (2–4 µg/mL) against *Salmonella enterica* ATCC 35664. Each value represents the mean ± standard deviation from three independent replicates. Asterisks represent the statistical differences from the values for cecropin A-treated cells (Student’s *t* test; * *p* < 0.05). # represents statical difference between mean values of HEWL/cecropin combinations containing 2 and 4 µg/mL cecropin A.

MIC analysis was conducted to assess the antibacterial activity of the lysozyme and cecropin A, and their combination against the Gram-negative *Salmonella enterica* ATCC 35664, in Muller Hinton broth. None of the agents showed complete bacterial growth inhibition (no measurable MIC) against *Salmonella enterica* ATCC 35664 within the tested concentrations, whereas HEWL displayed MIC of 500 µg/mL against *M. lysodeikticus.* Accordingly, HEWL with a final concentration of 500 µg/mL was used for the subsequent experiments. CecA alone showed a relative growth inhibitory activity against *Salmonella enterica* ATCC 35664 (Figure 3). For example, CecA inhibited *Salmonella enterica* ATCC 35664 growth by 29.59 ± 12.36% to 45.09 ± 1.1% (shown as turbidity reduction; Figure 3) using final concentrations of 2–20 µg/mL, respectively. We further investigated the potential synergy between HEWL and CecA by combing 500 µg/mL of HEWL with ascending concentrations of CecA (2–20 µg/mL). Although no measurable MIC was also detected in case of the HEWL/CecA combination against *Salmonella enterica* ATCC 35664, adding 500 µg/mL HEWL significantly improved (*p* < 0.05) the overall growth inhibition activity in a CecA dose-dependent manner within the CecA concentration range of 2–4 µg/mL. For example, adding 500 µg/mL HEWL to 2 and 4 µg/mL CecA caused a turbidity reduction of 50.12 and 59.17, instead of 29.59 and 33.01, respectively (Figure 3), suggesting a bacterial count reduction rather than complete eradication. Beyond 4 µg/mL, the observed turbidity reduction due to HEWL/CecA combination showed no statistical difference (*p* > 0.05, Figure 3). The additive inhibitory effect of HEWL predicts its possible passing through the cecropin A-permeabilized outer membrane and degrading the peptidoglycan layer.

**Figure 4:**
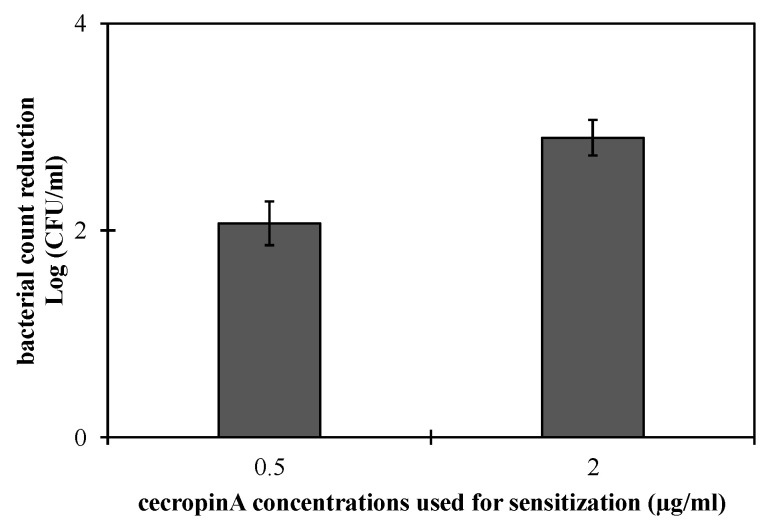
The effect of pre-treating bacterial cells with cecropin A on HEWL antibacterial activity. *Salmonella enterica* ATCC 35664 was grown till the exponential phase, pelted, and washed twice using 10 mM potassium phosphate buffer. The collected cells were then treated with 0.5 and 2 µg/mL cecropin A for 10 min and washed twice using the same buffer. The sensitized cells were then treated with 500 µg/mL HEWL lysozyme for 1 h at 35 °C. Log reduction in the bacteria count was calculated with respect to the pre-treated cells exposed to buffer instead of HEWL.

This assumption was further investigated by the sequential treatment of *Salmonella enterica* ATCC 35664 in an exponential growth phase with CecA (0.5 and 2 µg/mL) for 10 min followed by 500 µg/mL HEWL for 1 h (Figure 4). Treating bacterial cells with 500 µg/mL HEWL showed bacterial log count reductions of 2.06 ± 0.21 and 2.89 ± 0.17 when the cells were pre-treated with 0.5 and 2 µg/mL CecA, respectively. This observation corroborates our hypothesis of the outer membrane permeabilizing action of CecA.

### 3.3. The Combination of Cecropin A and Lysozyme Is Effective against Bacteria in the Exponential and Stationary Growth Phases

**Figure 5:**
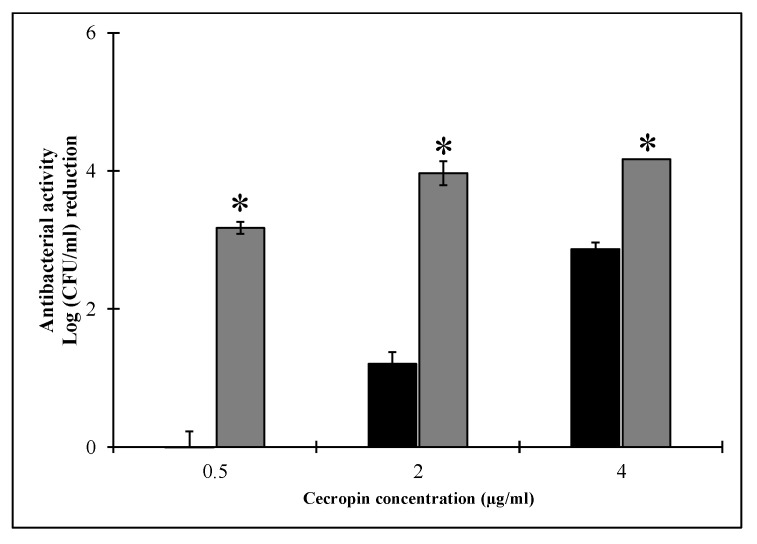
The antibacterial activity of HEWL (500 µg/mL) in combination with cecropin A (grey bars) against exponentially grown *Salmonella enterica* ATCC 35664. Black bars represent controls conducted by challenging bacterial cells to three ascending concentrations of cecropin A. LB exponentially grown bacterial cells were pelted, diluted to 10^6^ CFU/mL and resuspended in 10 mM potassium phosphate buffer. The prepared culture was then subjected to a combination of 500 µg/mL HEWL with escalating doses of 0.5, 2 and 4 µg/mL of cecropin A. A parallel experiment was conducted by treating the prepared culture with the same doses of cecropin A. Negative controls were performed using 10 mM potassium phosphate buffer instead of protein. Each value represents the mean ± standard deviation from three independent replicates. Asterisks represent the statistical differences from the values for cecropin A-treated cells (Student’s *t* test; * *p* < 0.05).

The antibacterial activity of HEWL was further investigated in the presence of three escalating doses of CecA (0.5, 2, 4 µg/mL) against both exponential and stationary grown *Salmonella enterica* ATCC 35664 suspended in 10 mM potassium phosphate buffer (pH 6.6). Bacteria in the logarithmic phase are generally sensitive to the action of PGHs [23]. However, in the current study, HEWL alone displayed no antibacterial activity (up to 1 mg/mL; data not shown) against exponentially grown *Salmonella enterica* ATCC 35664. Interestingly, HEWL reduced initial *Salmonella enterica* ATCC 35664 count (~10^6^ CFU/mL) by 3.17 ± 0.08 log units when CecA was added as low as 0.5 µg/mL (Figure 5). Moreover, increasing the CecA concentration to 2 µg/mL significantly (*p* < 0.05) improved the HEWL bactericidal effect around an additional one-log unit to be 3.96 ± 0.17 log units, while doubling the CecA concentration to 4 µg/mL lowered the challenged bacterial count below the detection limit (100 CFU/mL; corresponds to 4.16 log unit; Figure 5). CecA alone showed no direct antibacterial effect on exponentially grown *Salmonella enterica* ATCC 35664 at a concentration of 0.5 µg/mL. Nonetheless, higher concentrations decreased the challenged bacterial count by 1.20 ± 0.17 and 2.86 ± 0.09 log units when using 2 and 4 µg/mL, respectively (Figure 5). In conclusion, CecA as low as 0.5 µg/mL sensitized *Salmonella enterica* ATCC 35664 to the killing action of HEWL, whereas higher CecA concentrations exerted an additional antibacterial effect.

**Figure 6:**
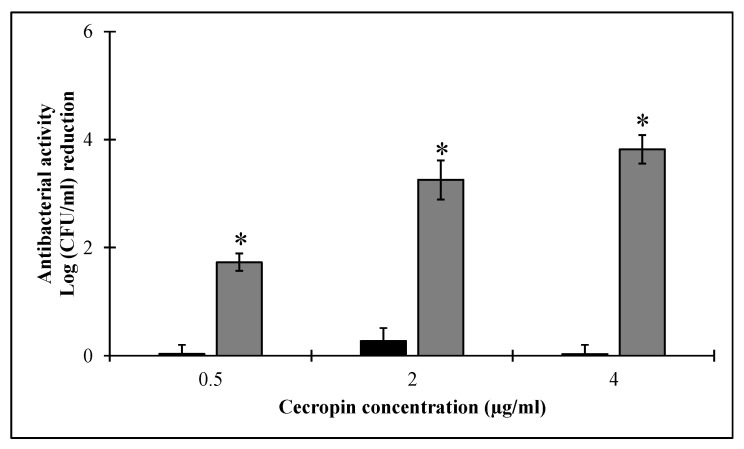
The antibacterial activity of HEWL (500 µg/mL) in combination with cecropin A (grey bars) against stationary grown *Salmonella enterica* ATCC 35664. Black bars represent controls conducted by challenging bacterial cells to three ascending concentrations of cecropin A. LB exponentially grown bacterial cells were pelted, diluted to 10^6^ CFU/mL and resuspended in 10 mM potassium phosphate buffer. The prepared culture was then subjected to a combination of 500 µg/mL HEWL with escalating doses of 0.5, 2 and 4 µg/mL of cecropin A. Negative controls were performed using 10 mM potassium phosphate buffer instead of protein. Each value represents the mean ± standard deviation from three independent replicates. Asterisks represent the statistical differences from the values for cecropin A-treated cells (Student’s *t* test; * *p* < 0.05).

Similarly, the previous combinations were evaluated against stationary grown *Salmonella enterica* ATCC 35664. The challenged bacteria were found refractory to the bactericidal effect of HEWL alone (final concentration up to 1000 µg/mL) and CecA (up to 4 µg/mL) when used individually. Nevertheless, combining 500 µg/mL HEWL with 0.5 µg/mL CecA statistically reduced (*p* < 0.05) the stationary grown bacterial cell count by 1.73 ± 0.16 log unit. The overall antibacterial activity of HEWL was further increased upon increasing CecA concentration in a dose-dependent manner with the activity of 3.18 ± 0.23 log in the presence of 4 µg/mL CecA (Figure 6).

### 3.4. Cecropin A/Lysozyme Combination Is Effective against Different Salmonella enterica Serovars

**Figure 7:**
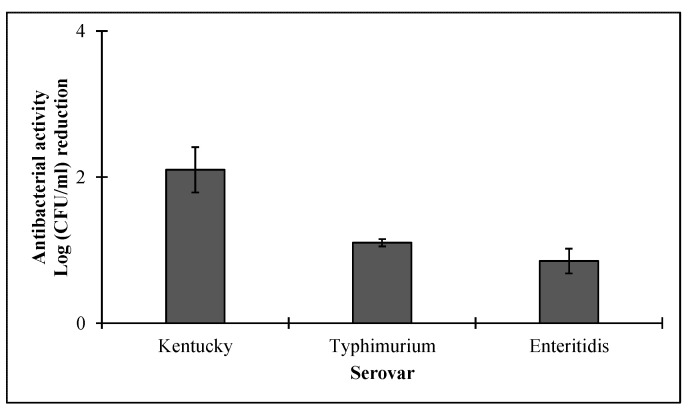
Serovar spectrum of Cecropin A/HEWL combination. LB exponentially grown *Salmonella enterica* serovars Kentucky, Typhimurium and Enteritidis were pelted, diluted to 10^6^ CFU/mL and resuspended in 10 mM potassium phosphate buffer. The prepared culture was then subjected to a combination of 500 µg/mL HEWL mixed with 0.5 µg/mL of cecropin A. Controls were performed using 10 mM potassium phosphate buffer instead of protein. Each value represents the mean ± standard deviation from three independent replicates.

The potential antibacterial activity of HEWL (final concentration of 500 µg/mL) combined with CecA (final concentration of 0.5 µg/mL) was tested against a panel of five *Salmonella enterica* different serovars isolated from duck, pigeon, and quail carcasses found in Egyptian market (Table 1). The isolated strains were found unresponsive to the 16 tested antibiotics [8]. The combination showed a serovar-dependent response (Figure 7). *Salmonella enterica subsp*. enterica serovar Kentucky was the most sensitive strain, being reduced by 2.1 ± 0.31 log units, whereas Typhimurium and Enteritidis serovars responded marginally (~one log unit reduction) to HEWL/CecA combination (Figure 7). On the other hand, *Salmonella enterica* serovars Hato and Shangani were found refractory to HEWL/cecropin (Data not shown).

### 3.5. Cecropin A Is Compatible with Oral Epithelial Cells within the Test Concentrations

**Figure 8:**
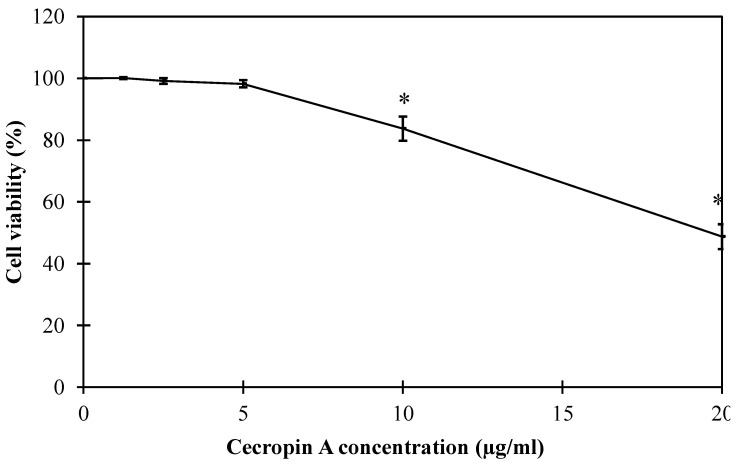
Change of cell viability of oral epithelial cell line treated with different cecropin A concentrations. The maintained cell line was treated with different cecropin A concentration (0–20 µg/mL) for 72 h. The treated cells were then fixed using 10% TCA and treated with SRB for 10 min for assessing cell viability spectrophotometrically at 540 nm. Each value represents the mean ± standard deviation from three independent replicates. Asterisks represent the statistical differences from the values for 10 mM tris buffer pH 7.5-treated cells (Student’s *t* test; * *p* < 0.05).

HEWL has been reported as a safe food preservative that is widely used in food applications [40]. However, CecA safety is controversial due to its reported cell membrane depolarizing activity [25]. Since our combination is proposed for food application, we evaluated CecA potential cytotoxicity against oral epithelial cells using SRB assay. CecA displayed no significant change (*p* > 0.5) in the overall cell viability up to 5 µg/mL. However, higher concentrations (≥10 µg/mL) significantly reduced cell viability by 83.7 ± 3.9 and 48.73 ± 4.05 at CecA concentrations of 10 and 20 µg/mL, respectively (Figure 8). Because the improvement of HEWL activity against *S. enterica* serovars was achieved with safe cecropin A concentrations (<5 µg/mL), the proposed combination is suggested to be safe for food application.

## 4. Discussion

In the current study, we evaluated the potential antibacterial activity of the natural HEWL in combination with antimicrobial peptide CecA against a panel of MDR *Salmonella enterica* prevalent serovars [8]. HEWL is a cheap, abundant, and safe lysozyme with reported antibacterial activity against Gram-positive pathogens. Therefore, it is widely used as a food preservative to control bacterial growth. However, its activity is diminished in Gram-negative bacteria due to the formidable outer membrane barrier. Therefore, the outer membrane must be breached to make lysozyme access to PG possible. Here, we virtually evaluated the outer membrane permeabilizing activity of cecropin A. CecA structural analysis revealed an α-helical peptide with the charged and hydrophobic amino acids distributed on the opposite site implying amphipathicity to CecA (Figure 1 and Figure S2). Amphipathic helices have been previously reported with outer membrane penetration ability rendering PGHs linked to their terminals effective against Gram-negative bacteria [19,23,24,26,39,40,41]. CecA interaction with the outer membrane was further virtually confirmed using the membrane–protein simulation model (Figure 2). The established simulation run presented the ability of CecA to interact with both the ionic and hydrophobic faces of the membrane model distorting its integrity (Figure 2A–C, supplementary video; https://zenodo.org/record/7058606#.YzNGQHbP2M8, accessed on 28 August 2022). We speculated that the predicted interaction of CecA peptide with *Salmonella enterica* outer membrane could facilitate the access of the exogenously applied HEWL and hence antibacterial activity. Upon elaboration, HEWL displayed no growth inhibitory action against *Salmonella enterica* ATCC 35664 grown in LB broth up to 1 mg/mL. However, combination with incremental concentrations of CecA caused bacterial turbidity reduction up to ~70% with 10–20 µg/mL CecA (Figure 3). This suggested a successful penetration of HEWL to the limitative outer membrane barrier and subsequent digestion of the PG layer.

Bacterial growth phase has a significant impact on the overall antibacterial activity of the applied PGH. In the logarithmic growth phase, bacteria are more sensitive, whereas the stationary grown phenotypes are found to be more refractory [23,42,43,44]. In the current study, we evaluated the antibacterial outcome of combining HEWL with ascending concentrations of CecA against bacteria in both stationary and logarithmic growth phases. Combining HEWL with 0.5 µg/mL CecA displayed a bacterial count reduction of 3.17 ± 0.08 log units (Figure 4) without individually noted activity for both agents. Increasing CecA concentration to 2 and 4 µg/mL in the combination improved the bactericidal activity of HEWL that is presented as bacterial count reduction of 3.96 ± 0.17 and 4.16 log units, respectively. Noteworthy, 2 and 4 µg/mL CecA had a direct antibacterial activity on the exponentially grown *Salmonella enterica* ATCC 35664 (Figure 5). Direct antibacterial activity of cecropins were reported before against different Gram-negative bacteria [25,44]. Cecropins exert their direct antibacterial effect by interacting with the cytoplasmic membrane leading to membrane depolarization and subsequent bacterial death [25]. Therefore, the lack of CecA antibacterial activity at low concentrations (as low as 0.5 µg/mL) could be explained by the entrapment of all CecA molecules in the outer membrane leading to its permeabilization without measured effect on the cytoplasmic membrane. Therefore, we observed the antibacterial effect only in combination with HEWL. However, increasing the CecA concentration would make more molecules available for interaction with the inner membrane. In the case of stationary grown bacterial cells, CecA showed no antibacterial effect up to 4 µg/mL, suggesting lowering the outer membrane permeability that calls for more CecA for permeabilizing. This explained the relatively lower antibacterial activity achieved upon combining 0.5, 2, and 4 µg/mL with 500 µg/mL HEWL compared to exponentially grown cells (Figure 5).

Different approaches have been pursued to permeabilize the outer membrane of the Gram-negative bacteria, including the application of high hydrostatic pressure [15,45], using outer membrane permeabilizer, conjugation with dextran [17], and enzyme denaturation [46]. EDTA-induced permeabilization of the outer membrane was commonly reported, especially with peptidoglycan hydrolases from phage origin [35,45,47,48,49,50]. For example, Liu et al. reported on a phage-derived peptidoglycan hydrolase (LysWL59) that displayed antibacterial activity against *S. typhimurium* with the aid of 0.5 mM EDTA [50]. Nevertheless, another report observed a lack of antibacterial activity of phage-derived PG degrading enzyme and HEWL against *Salmonella typhimurium* LT2 even in the presence of 0.5 mM EDTA [19]. Here, our test combination was found effective against three prevalent *Salmonella enterica* serovars, including Typhimurium (Figure 6). The net antibacterial activity of PHGs depends mainly on the capability of the enzyme to penetrate the outer membrane and their access to PG. EDTA permeabilizes the outer membrane by chelating the stabilizing divalent cations (Ca^2+^, Mg^2+^) thus interfering only with the stabilizing ionic forces. However, in the current study, we used the CecA amphipathic helix peptide that interferes with both ionic and hydrophobic forces [20], suggesting a better permeabilizing activity as shown in both stationary and exponentially grown cells.

HEWL is an abundant protein that is naturally present at high concentrations in many food products, especially egg and milk. Moreover, it has been approved as a safe food additive and preservative [12]. Therefore, we excluded it from safety assessment. On the other hand, CecA could interact with the cytoplasmic membrane due to its amphipathic nature, suggesting a possible cytotoxicity. Studies have reported the cytotoxic effect of cecropin against human cancer cell lines making its safety assessment crucial for safe consumption [51,52]. Therefore, the potential cytotoxicity of CecA was assessed using an oral epithelial normal cell line. CecA displayed no cytotoxicity against the oral epithelial cell line up to 5 µg/mL. However, higher concentrations showed statistically lower cell availability indicating cytotoxicity (Figure 8). These observations suggest safe oral consumption of CecA with concentration <5 µg/mL. Here, we have proved experimentally that CecA could be used as low as 0.5 µg/mL to improve the antibacterial activity of HEWL (Figure 7), making our combination suitable for application. Other criteria that should be investigated in the future include the effect of this combination on oral microbiome and immunity. Mere use of cecropins has shown a bad effect on the oral microbiome, including changes in the diversity and richness of rat oral microbial communities and the relative abundance of the pathogen *Acinetobacter baumannii* [53]. Since our proposed combination depends mainly on the antibacterial activity of HEWL in the presence of small concentrations of CecA, as low as 0.5 µg·mL, the adverse effect on the oral microbiome is suggested to be lower.

## 5. Conclusions

HEWL is an abundant, natural, and safe peptidoglycan hydrolase with reported antibacterial activity against Gram-positive pathogens. Nevertheless, the protective outer membrane barrier limits its activity against prevalent Gram-negative food pathogens such as *Salmonella enterica*. Based on previous reports and in silico structural analysis, CecA extended the spectrum of HEWL to cover different *Salmonella enterica* serovars via interference with the outer membrane stabilizing forces, thus making access of HEWL to peptidoglycan layer possible. Moreover, the possible cytotoxicity of CecA towards oral epithelial cells was excluded indicating its safety for oral consumption. Accordingly, we present HEWL/CecA as a cheap, effective, safe, and sustainable approach for combating the challenging *Salmonella enterica serovars*.

## Figures and Tables

**Table 1:** List of *Salmonella enterica* serovars used in the current study. Except for the reference strain, all Salmonella serovars were previously isolated, identified, and previously tested for antibiotic sensitivity [8].

*Salmonella enterica* Serovars	Carcasses Source	Antibiotic Resistance ^a^	Resistance Genes
Typhimurium	Duck	DA, S, TE, NA, AK, SXT, AMP, K CTX, CT, CN, CP, C, MEM, LEV	blaCMY-1, blaOXA-2
Kentucky	Duck	DA, S, TE, NA, AK, SXT, AMP, K, CTX, CT, CN, CP, C, MEM, LEV	blaCMY-1
Enteritidis	Quail	DA, S, TE, NA, AK, SXT, AMP, K, CTX, CT, CN, CP, C	blaCMY-1, blaOXA-2
Hato	Pigeon	Not tested	Not tested
Shangani	Quail	DA, S, TE, NA, AK, SXT, AMP, K, CTX, CT, CN, CP	blaCMY-1, blaCMY-2
*Salmonella enterica* ATCC 35664	-	-	-

^a^: DA = Clindamycin, S = Streptomycin, TE = tetracycline, NA = Nalidixic acid, AK = amikacin, SXT = Trimethoprim/Sulphamethoxazole, AMP = Ampicillin, K = Kanamycin, C = chloramphenicol, CTX = cefotaxime, CT = colistin, CN = Gentamicin, CP = Ciprofloxacin, MEM = Meropenem, LEV = Levofloxacin.

## Data Availability

Not applicable.

## Supplementary Materials

The following supporting information can be downloaded at: https://www.mdpi.com/1999-4923/14/10/2201/s1, Figure S1: Ramachandran plot of the generated cecropin model. All the protein residue are plotted inside the high confidence α-helix region indicating a good quality of the model; Figure S2: Helical-wheel projection cecropin A amphipathic helix.
