# Supplementary material for: Cecropin A Improves the Antibacterial Activity of Hen Egg White Lysozyme against Challenging Salmonella enterica Serovars
Source: Pharmaceutics. 2022 Oct 16;14(10):2201. doi: 10.3390/pharmaceutics14102201 (PMC9610619; doi:10.3390/pharmaceutics14102201)
Supplement: Supplementary file 1 [file pharmaceutics-14-02201-s001.zip › pharmaceutics-1932290-supplementary.pdf]

Supplementary Material

Cecropin A Improves the Antibacterial activity of Hen Egg White Lysozyme against challenging *Salmonella enterica* serovars

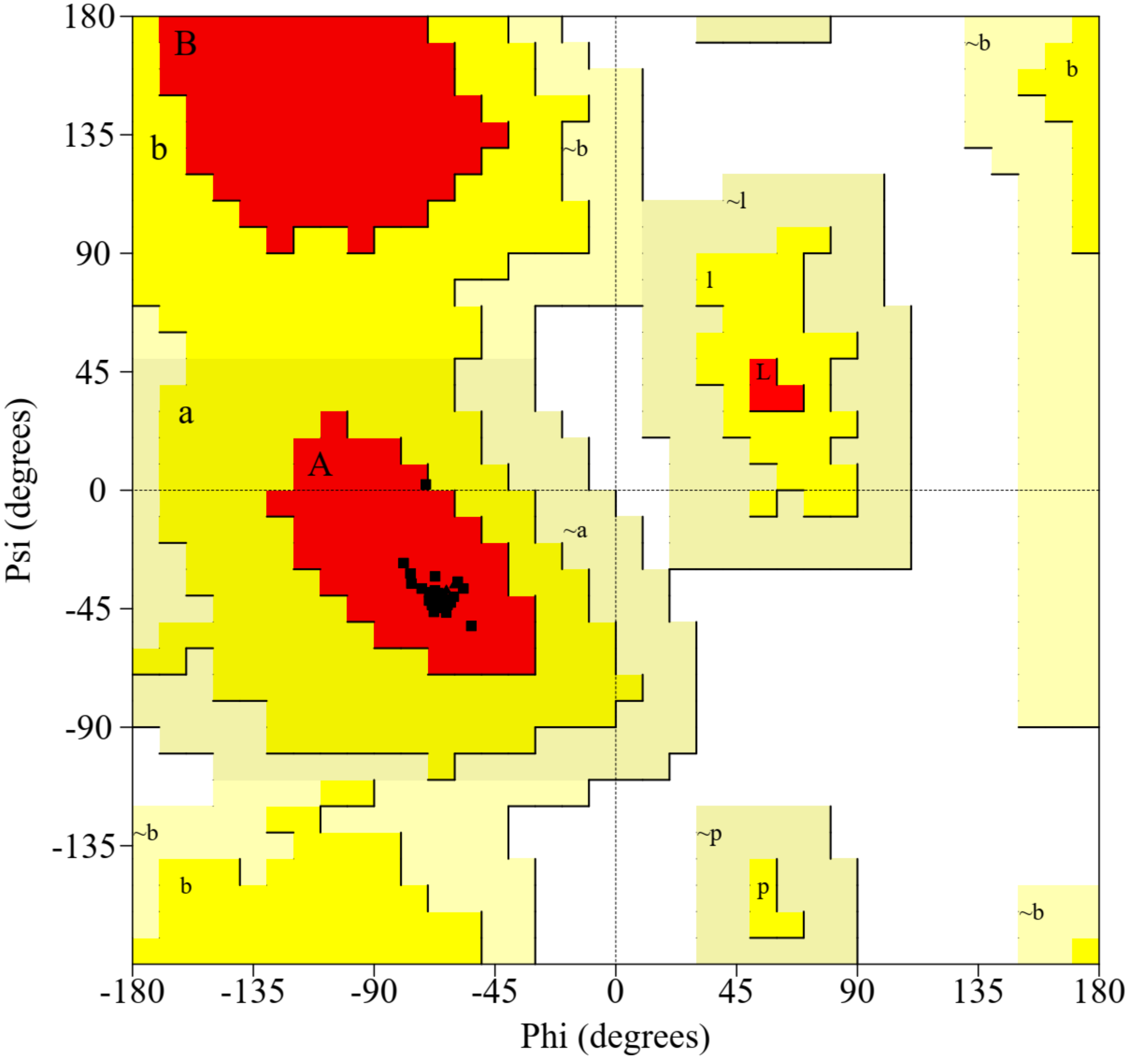

Figure S1. Ramachandran plot of the generated cecropin model. All the protein residue are plotted inside the high confidence α-helix region indicating a good quality of the model.

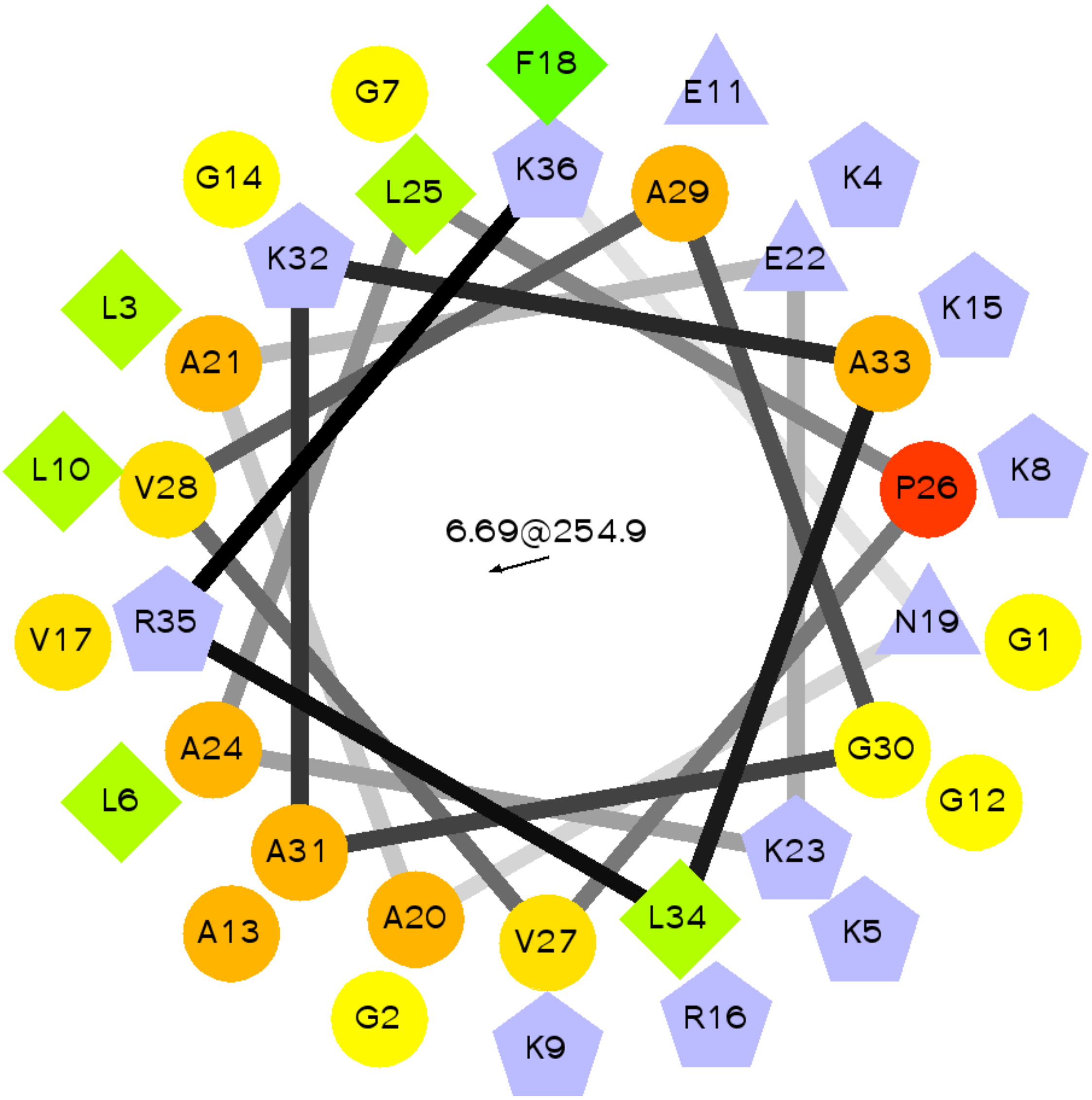

Figure S2. Helical-wheel projection cecropin A amphipathic helix. Charged amino acid (light purple) are concentrated on the right side, whereas the non-charged counter parts are dominating the left side.
